# Isolation of indigenous *Bacillus velezensis* from aging tobacco leaves for improving the flavor of flue-cured tobacco

**DOI:** 10.3389/fmicb.2025.1623279

**Published:** 2025-07-16

**Authors:** Xiao-Jie Shan, Lifeng Jin, Feng Li, Sheng-Bing Yang, Xiao-Juan Zhang, Li-Juan Chai, Jin-Song Gong, Jin-Song Shi, Zhen-Ming Lu, Zong-Yu Hu, Zheng-Hong Xu

**Affiliations:** ^1^Key Laboratory of Industrial Biotechnology of Ministry of Education, School of Biotechnology, Jiangnan University, Wuxi, China; ^2^Zhengzhou Tobacco Research Institute of CNTC, Zhengzhou, China; ^3^National Engineering Laboratory of Cereal Fermentation and Food Biomanufacturing, Jiangnan University, Wuxi, China; ^4^School of Life Sciences and Health Engineering, Jiangnan University, Wuxi, China; ^5^China Tobacco Jiangsu Industrial Co., Ltd., Nanjing, China; ^6^Innovation Center for Advanced Brewing Science and Technology, College of Biomass Science and Engineering, Sichuan University, Chengdu, China

**Keywords:** flue-cured tobacco, bacterial community, *Bacillus velezensis*, bioaugmentation, aging

## Abstract

**Introduction:**

Aging of flue-cured tobacco is a slow microbial fermentation process that usually lasts for 2–3 years, which plays an important role in improving the quality of final products.

**Methods:**

Re-dried tobacco leaves from seven Chinese regions were subjected to a controlled aging environment for 12 months. The bacterial community succession and volatile compounds dynamics in tobacco leaves throughout aging process were monitored. The main functional microorganisms were isolated from the tobacco leaves and inoculated into lower-grade tobacco leaves to evaluate their metabolic functions.

**Results:**

Spearman’s rank correlation analysis revealed that *Bacillus* was a key genus driving aroma production. By using the pure culture method, strain *Bacillus velezensis* TB-1 with the best enzyme-producing capacity was screened from high-grade tobacco leaves. Furthermore, *Bacillus velezensis* TB-1 was applied to low-grade tobacco leaves for solid-state fermentation. GC-MS results showed a significant increase in volatile compounds in fermented tobacco leaves compared to unfermented controls. The contents of 2-methoxy-4-vinylphenol (clove and wood flavors), megastigmatrienone (tobacco and spicy aromas), 2-methyl-hexanoic acid (cream flavor) increased significantly. Sensory evaluation confirmed that fermentation with *Bacillus velezensis* TB-1 markedly enhanced tobacco leaf flavor quality.

**Discussion:**

This study identified *Bacillus velezensis* as a core aroma-producing microorganism in tobacco aging, demonstrating its application for fermentation to enhance leaf quality, thus establishing a novel strategy for tobacco improvement.

## 1 Introduction

Tobacco is the most widely cultivated economic crop in the world. China, as a major tobacco producer, particularly in flue-cured tobacco, has approximately over 300 million smokers, accounting for about 25% of the world’s smokers; China’s tobacco production accounts for 35% of the world’s total, and its tobacco sales account for 32% (Cui, 2021). The environment and climate in tobacco-growing areas largely determine the quality of tobacco cultivation (Hu et al., 2022). Additionally, aging and fermentation methods, as well as the role of microorganisms, endow tobacco leaves with more flavors (Qiao et al., 2016; Li et al., 2019).

In the traditional brewing industry, fermentation is an important process to improve food quality, including fermented wine, fermented meat, tea and so on (Cai et al., 2020; Li et al., 2023; Jin et al., 2024). Aging treatments can intensify flavors and reduce irritation in ingredients. However, spontaneous aging is time-consuming and thus less efficient. Currently, the application of accelerating the aging process through manual intervention to reduce the time for improving food quality is on the rise (Guan et al., 2020). For instance, tobacco fermentation can be categorized into two methods: natural fermentation and accelerated fermentation. Natural fermentation involves storing re-dried tobacco leaves in a warehouse. Under the influence of natural climate variations, the inherent quality of the tobacco leaves changes. This process typically takes 2–3 years. Accelerated fermentation entails placing fresh tobacco in a fermentation room with controlled temperature and humidity to accelerate the aging of tobacco leaves and improve the quality of raw materials in a short period. Therefore, understanding the microbial changes at different stages of the accelerated aging of tobacco leaves is crucial. Analyzing the dynamic changes of microorganisms during the accelerated mellowing process facilitates the subsequent artificial utilization of microorganisms to optimize the mellowing process (Liu et al., 2021; Wang et al., 2022; Wu et al., 2022). Analyzing the metabolic functions of these microorganisms can deepen our understanding of the essential chemical reactions during aging, thereby improving the quality of tobacco leaves (Zhao et al., 2007; Huang et al., 2010).

Tobacco leaf aging is a crucial process for influencing and enhancing the quality of flue-cured tobacco. In recent years, the application of microbial technology in accelerated tobacco leaf fermentation has witnessed rapid development (Civilini et al., 1997; Wang et al., 2012; Weng et al., 2024). For instance, the aroma development of cured tobacco leaves can be promoted by isolating and applying *Bacillus subtilis* from fermented tobacco leaves (Ma et al., 2024). Applying a co-cultivation of *Bacillus amyloliquefaciens* and *Bacillus kochii* to tobacco leaves was found to increase the sugar-base ratio to nearly 10 and enhance the tobacco aroma (Wu et al., 2021). During the fermentation process, starch-degrading strains of *Paenibacillus amylolyticus* were identified and isolated from high-quality tobacco leaves. When this strain was applied to low-grade tobacco leaves, the quality of the tobacco leaves improved significantly (Gong et al., 2023).

In this study, tobacco leaves from seven distinct production areas were aged for up to one year under controlled conditions, with temperatures ranging from 25 to 30°C and humidity between 60 and 65%. Firstly, the correlation analysis between microbial community succession in tobacco leaves and volatile compound changes during fermentation was conducted to predict functional bacteria involved in flavor formation. Secondly, enzyme-producing functional bacteria were isolated and identified from high-grade aged tobacco leaves using pure cultures. Subsequently, the enrichment of functional microorganisms was applied to the aging process of low-grade tobacco leaves to enhance their flavor quality.

## 2 Materials and methods

### 2.1 Experimental materials

The main tobacco species cultivated and grown in China is *Nicotiana tabacum* L. Due to natural evolution and artificial breeding, *Nicotiana tabacum* L has evolved into different varieties. In this study, four varieties of tobacco leaves were sampled in 2021 from seven key tobacco-producing areas in China (Supplementary Table S1). The seven key tobacco-producing areas included Lijiang in Yunnan Province, Zunyi in Guizhou Province, Chenzhou in Hunan Province, Liangshan in Sichuan Province, Sanming in Fujian Province, Sanmenxia in Henan Province, and Linyi in Shandong Province. There were four tobacco varieties in the sampled tobacco leaves, including Yunyan87, CB-1, Qinyan96, and Zhongyan Texiang301 (Supplementary Table S1). Accelerated aging experiment of re-dried tobacco leaves was conducted under controlled temperature and humidity conditions of 25–30°C and 60–65%, respectively (Guan et al., 2020; Supplementary Figure S1). The test duration was 0–12 months, with monthly sampling. For each sample preparation, about 350–400 g tobacco leaves were excised from the four corners of tobacco box and subsequently mixed. During the aging process, the appearance quality of tobacco leaves showed that the color gradually deepened, the saturation and uniformity slightly increased, the oil content of tobacco leaves slightly increased, and the luster gradually darkened. With the increase of aging time, the leaf shape of tobacco was more contracted, and the dry matter of leaves was reduced (Supplementary Figure S1). In this study, all the fermented tobacco samples were provided from Jiangsu China Tobacco Industry Co., Ltd., and Yunnan Lijiang C2F tobacco aged for 2 years was used as the strain separation material, and Low-quality C3F tobacco aged for 2 years from Sanmenxia, Henan Province was used as the inoculated tobacco.

Isolation medium: 20 g glucose, 2 g peptone, 2 g yeast extract, 0.5 g magnesium sulfate heptahydrate, 1 g dipotassium dihydrogen phosphate, 0.46 g potassium dihydrogen phosphate, tobacco powder 2 g, distilled water 1,000 mL. Autoclave at 115°C for 20 min. Tobacco powder is obtained by crushing tobacco leaves and passing through a 20-mesh screen. LB medium: Yeast extract 5 g, peptone 10 g, sodium chloride 10 g, distilled water 1,000 mL. Autoclave at 121°C for 20 min. Solid medium added with 20 g (per 1,000 mL medium) of agar.

### 2.2 DNA extraction and high throughput sequencing

According to the preliminary experimental results of this study, tobacco leaf microbial genomic DNA was extracted by *in situ* extraction, treatment with protease K and RNase A, and kit Kit2 extraction. The specific procedure is as follows: Prepare five 96-well deep-hole plates and add 600 μL buffer bead binding solution, 20 μL protease K, 5 μL RNase A, respectively. 700 μL wash buffer 1,700 μL wash buffer 2,700 μL wash buffer 3,100 μL elution buffer. Then, 100–200 mg ground tobacco leaves powder were taken into the centrifugal tube with grinding beads, and 1 mL buffer ATL/PVP-10 was added. After grinding the samples on a high-speed grinder, the tube was incubated at 65C° for 20 min. Then, centrifuged at 10,000 rpmfor 5 min, and transferred the supernatant to a new centrifuge tube, add 0.6 mL Buffer PCI, vortex mix 15 s. 12,000 rpm centrifuge 10 min, transfer the supernatant to a deep-hole plate with magnetic bead binding solution. Start Kingfisher to select the corresponding program, put each deep-hole plate in the corresponding position of the instrument, and run the program. At the end of the procedure, the DNA solution in the elution buffer deep-hole plate was transferred to a 1.5 mL centrifuge tube for preservation. DNA concentration and purity were measured using Nanodrop 2,000 (Thermo Fisher Scientific, United States), and qualified DNA was sent to BGI for Illumina MiSeq amplicon sequencing (2 × 300 bp). Primers 338F (5′-ACTCCTACGGGAGGCAGA-3′) and 860R (5′-GGACTACHVGGGTWTCTAAT-3′) were used to amplify the V3-V4 region of bacterial 16S rRNA (Caporaso et al., 2010; Zhou et al., 2020).

### 2.3 Genome assembly, classification, and functional annotation

The sequencing work of this study was undertaken by BGI (BGI Genomics Co., Ltd., Shenzhen, China). After sequencing, the clean data underwent quality assessment, including quality control, filtering, and analysis of the raw sequences, all in accordance with the standard operating procedures of QIIME. The main processes include:

(1) Quality control: Conduct quality control and sample splitting on clean data, remove low-quality sequences (length less than 150 bp, fuzzy base N, sequence quality score less than Q20) through split analysis, then use Usearch software to remove chimera sequences, and finally obtain valid sequences.
(2) ASV clustering: ASV (Amplicon Sequence Variant, ASV) is based on the sequence to de-noise, exclude or correct the error sequence, and select the trusted sequence with high abundance as the representative sequence. After removing the error sequence, set the Identity criteria to 100% for clustering (Callahan et al., 2017).
(3) Taxonomic analysis: Representative ASV sequences were compared with the bacterial 16S rRNA gene database Greengenes (13.8 edition). According to Genbank^1^ sequence, through further analysis of ASV Megablast level annotation information sequence of species.
(4) Diversity analysis: All samples of ASV were diluted to the same sequencing depth based on the sequencing depth of bacterial and fungal samples respectively, and then α diversity and β diversity analysis were performed (Fierer and Jackson, 2006; Su et al., 2011; Su et al., 2017).

### 2.4 Analysis of volatile compounds in tobacco leaves

Volatile compounds of tobacco leaves were determined by GC-MS (model 7890B-5977A, Agilent, Palo Alto, CA, United States) as follows: 0.5 g tobacco leaves powder was taken and 6 mL saturated NaCl solution was added. 2-Octanol (10 μL, 20 mg/L, dissolved in chromatographic grade methanol) was added as the internal standard. PAL 3 autosampler (CTC Analytics AG, Switzerland) with SPME Fiber (thickness 80 μm, length 10 mm, DVB/C-WR/PDMS) (Agilent Technology Co., Ltd., United States) was used to extract the volatile compounds from the headspace of the sample. The sample was preheated at 50°C for 5 min and then extracted for 30 min at 50°C. After extraction, insert the extraction head into the gas chromatographic mass spectrometer for analysis (250**°C**, 5 min). Chromatographic conditions: DB-WAX capillary column (column length 60 m, inner diameter 0.32 mm, liquid film thickness 0.25 μm) was used. The temperature of the inlet was 250**°C**, and the heating procedure was 50°C. After 2 min, the temperature was raised at 3°C/min to 145°C, and then at 15°C/min to 230°C, and the temperature was maintained for 3 min. Helium is used as the carrier gas, the carrier gas flow rate is 1.0 mL/min, and the shunt ratio is 20:1. Mass spectrum conditions: EI ionization source, ion source temperature is 260**°C**, electron bombardment energy: 70 eV, scanning mass range: 33–350 m/z. Internal standard method was used to calculate the content, and the relative content of each substance was obtained by comparing the peak area value (Ni et al., 2019; Yan et al., 2022; Yang et al., 2024).

### 2.5 Isolation of *Bacillus* species

A correlation analysis was performed to examine the relationship between the succession of microbial communities and alterations in volatile compound profiles during the aging of tobacco leaves, with the objective of identifying the species of functional microorganisms (Law et al., 2016). During the aging process of tobacco leaves, variations in multiple aroma volatile compounds necessitated the application of Fisher’s transformation to derive the mean correlation coefficient (Fisher, 1915). This method was employed to elucidate the correlation between individual bacterial genera and the overall flavor profile of the tobacco leaves.

To isolate *Bacillus* strains, 10 g tobacco leaves were cut into pieces and added to the 90 mL separation medium for enrichment for 48 h. The strain in tobacco leaf was separated by dilution coating method and purified by scribing. The isolated strains were cultured in LB medium at 37°C for 24 h, DNA was extracted to identify the strains, and finally cultured for fermentation and preservation (Fan et al., 2017; Mao et al., 2022; Cai et al., 2022).

### 2.6 Determination of enzyme activity in fermented tobacco leaves

A total of 10 strains including TB-1 were cultured in LB liquid medium at 37°C and 200 rpm for 24 h. The bacterial suspension was centrifuged at 10,000 rpm and 4°C for 5 min by high-speed freezing centrifuge (Eppendorf AG, Hamburg, Germany) to obtain the supernatant to determine the enzyme producing ability of the strain (Ning et al., 2021; Rabbee et al., 2019).

Defined 1 U of amylase activity was the amount of enzyme produced in 1 μg glucose of 1 mL enzyme per minute under the measurement condition at 35°C.

Defined 1 U of cellulase activity was the amount of enzyme produced in 1 μg glucose of 1 mL enzyme per minute under the measurement condition at 50°C.

Defined 1 U of pectinase activity was the amount of enzyme produced in 1 μg glucose of 1 mL enzyme per minute under the measurement condition at 50°C.

Defined 1 U of protease activity was the amount of enzyme hydrolyze casein and produces 1 μg tyrosine of 1 mL enzyme per minute under the measurement condition at 40°C.

Amylase, cellulase and pectinase were determined by DNS method. Briefly, 1% soluble starch, 1% sodium carboxymethyl cellulose and 0.5% pectin were dissolved in 0.1 M pH = 5.6 citric acid buffer. Add 1.0 mL of substrate to 1.0 mL of fermentation supernatant, shake well, place in the water bath at the corresponding temperature for 30 min, immediately add 2 mL of DNS solution, place the reaction solution in boiling water for 5 min for color reaction, and inactivate the enzyme. After cooling the solution, dilute it to 10 mL with distilled water, and then measure the absorbance at 540 nm with Spark Multimode Microplate Reader^®^ (Tecan Trading AG, Switzerland). The glucose standard curve showed that the glucose concentration showed a good linear relationship in the range of 0.1∼0.7 mg/mL, and the blank control was inactivated fermentation supernatant (Ma et al., 2024; Zhang et al., 2006; Wang et al., 2007).

Protease activity assay was using the forint phenol method referred to National standard National Food Industry Standardization Technical Committee (SAC/TC 64) (2023). The details of the assay were as follows. Adding 1 mL crude enzyme solution into 1 mL 10 mg/mL casein solution (prepared with 0.1 M pH = 7.5 phosphate buffer), and then incubating at 40°C of water bath for 10 min, followed by adding 2 mL 0.4 M trichloroacetic acid solution and standing for 20 min. Filter with 0.22 μm filter and take out 1 mL filtrate, add 5 mL 0.4 M Na**_2_**CO**_3_** and 1 mL forint reagent, then develop color in 40°C water bath for 20 min. Measure the absorbance value at 680 nm.

### 2.7 Fermentation of tobacco leaves

*Bacillus* sp. TB-1 was cultured in LB liquid medium at 200 rpm at 37°C for 24 h. The fermentation solution is evenly sprayed on the surface of the tobacco leaf at a ratio of 20% (v/w). The inoculated bacterial tobacco leaves were fermented for 48 h at 37C° at constant temperature and humidity of 80%RH (relative humidity) (Chen et al., 2016; Ma et al., 2024). The blank samples were treated with equal amounts of sterile distilled water and fermented under the same conditions. After fermentation, part of the tobacco sample is crushed through a high-speed crusher and filtered through a 20-mesh screen before being prepared for analysis. The other part of the tobacco leaves is completely stored in the refrigerator at 4C°.

### 2.8 Sensory evaluation of fermented tobacco leaves

Referring to the sensory evaluation index of quality style characteristics of flue-cured tobacco leaves (Luo et al., 2019; Zhao et al., 2025), the aroma style characteristics of TB-1 fermented tobacco leaves and unfermented control tobacco leaves were determined by sensory evaluation. Twelve healthy males who have received smoking training, aged between 25–40 years old, with experience in aroma description and intensity rating, and certified to evaluate cigarettes for sensory purposes were recruited for sensory evaluation. The sensory evaluation test was performed at room temperature (24°C) with a humidity of 55%. The evaluation was performed in accordance with the industry standard National Tobacco Standardization Technical Committee Cigarette Sample Technical Committee (SAC/TC 144/SC 10) (2015). Specifically, the intensity of the aroma is scored as 0–5points, of which 0–1 points is not too slightly pronounced, 2–3 points is slightly to fairly pronounced, and 4–5 points is more pronounced than pronounced. In addition, in order to further clarify the volatile compounds that affect the flavor of tobacco leaves, 8 volatile compounds that significantly increased after fermentation were selected based on GC-MS results.

Through sensory test, 8 volatile compounds were added at corresponding concentrations, including 2-methoxy-4-vinylphenol, megastigmatrienone, etc., to analyze their effects on tobacco flavor. Adjust the concentration of volatile compounds to the same level as in fermented tobacco leaves based on GC-MS results. Different tobacco aroma mixture models were prepared by adding a single key compound. After that, the differences between each addition model and the control tobacco model were evaluated by triangle test. Significance analysis: when 8 people answered correctly, the data was significant (*P* ≤ 0.05); When 9–10 people answered correctly, the data were highly significant (*P* ≤ 0.01); When 11–12 people answered correctly, the data was considered very significant (P ≤ 0.001) (Yang et al., 2024).

The sensory quality of fermented tobacco cigarettes was evaluated by China Tobacco Jiangsu Industrial Co., Ltd. The assessment panel comprised 7 qualified professional assessors. According to the Chinese tobacco industry recommended standards GB 5606.4-2005 [National Tobacco Standardization Technical Committee (TC 144), 2005], the sensory quality of FCT samples were evaluated using eight evaluation indexes, including aroma quality, aroma quantity, impurity, irritation, concentration, intensity, sweetness, aftertaste, fineness. The score of sensory evaluation was the sum of all indicator scores (Mao et al., 2022).

### 2.9 Statistical analyses

There were at least three parallels for each group of samples and the results were expressed as mean ± standard deviations. Principal component analysis (PCA) and partial least squares discrimination analysis (PLS-DA) are performed using SIMCA software (version 14.1, Umetrics, Sweden). Spearman correlation analysis of microorganisms with metabolites using Origin (version 2024b, OriginLab, United States). Significance difference analysis and One-way ANOVA were performed using SPSS (version 27.0, SPSS Inc., United States). Phylogenetic and molecular evolutionary analyses were conducted using MEGA version 11 (Tamura et al., 2021). Further statistical analysis and graphics were performed in EXCEL 2017 software (Microsoft Office, United States) and GraphPad Prism Software (version 8.0, GraphPad Software, United States).

## 3 Results

### 3.1 Succession of bacterial communities in tobacco leaves during aging process

**FIGURE 1:**
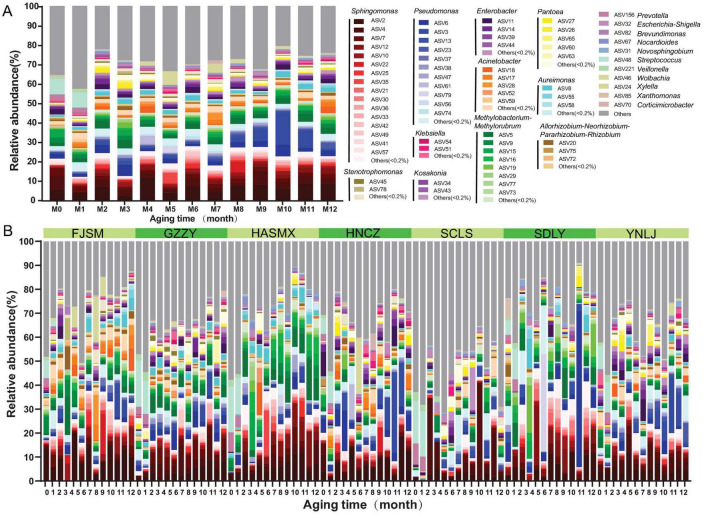
Succession of bacterial communities during the accelerated aging process of tobacco leaves under controlled environmental condition (temperature, 25–30°C; humidity, 60–65%). Species with an unannotated classification and a relative abundance below 0.5% in the sample were grouped into the “others” category. **(A)** Succession of bacterial communities within all tobacco leaf samples. **(B)** Succession of bacterial communities during the aging process of tobacco leaves originated from seven tobacco-producing regions. YNLJ, Lijiang in Yunnan province; GZZY, Zunyi in Guizhou province; HNCZ, Chenzhou in Hunan province; SCLS, Liangshan in Sichuan province; FJSM, Sanming in Fujian province; HASMX, Sanmenxia in Henan province; SDLY, Linyi in Shandong province.

Succession in bacterial communities within all tobacco leaf samples was observed over a 1-year accelerated aging period at the ASV level (Figure 1A), as well as the genus and phylum levels (Supplementary Figure S2). Throughout the aging process, the bacterial community was predominantly composed of Proteobacteria (86.2% average relative abundance), Firmicutes (5.7%), Bacteroidota (2.5%), and Actinobacteriota (3.2%) after 12 months. Across all samples, a total of 11,666 bacterial ASVs representing bacterial species were detected. These ASVs were classified into 1,009 genera (Figure 1A). *Sphingomonas*, *Pseudomonas*, *Methylobacterium*, and *Acinetobacter* were the dominant bacterial genera (Figure 1A). The Chao1, Sob and Ace indices of bacterial communities gradually increased with aging time, indicating an increase in species richness (Supplementary Figure S3). However, the Shannon and Simpson indices did not show significant difference among different aging time (*P* > 0.05), indicating the community structure remained relatively stable (Supplementary Figure S3).

The structure of the bacterial community in tobacco leaves from different production areas varied, yet the dominant species remained similar (Figure 1B). The majority of ASVs with high abundance are primarily classified into *Sphingomonas*, *Pseudomonas*, *Methylobacterium*, *Acinetobacter*, and *Enterobacter*. Among these, the abundance of *Sphingomonas* typically exhibits an initial increase followed by a decrease. In the FJSM region, for instance, ASV10 increased from 0.3 to 7.3% at M3, subsequently fluctuating and decreasing to 0.5% by M12. Similarly, in the HNCZ region, ASV2 rose from 4.0 to 14.2% at M4, before decreasing to 2.2% by M10. The abundance of *Pseudomonas* gradually increases over the aging period. For example, in the GZZY region, the relative abundance of ASV6 increased from 2.4% at M0 to 3.7% by M12. Overall, following 12 months of aging, the relative abundance of other species decreased compared to the initial stage, leading to a more uniform distribution of dominant ASVs.

Variance analysis revealed that the genera showing significant differences in relative abundance across different accelerated age stages were *Sphingomonas, Pseudomonas, Methylobacterium, Acinetobacter, Enterobacter, Aureimonas, Pantoea, Stenotrophomonas, Allorhizobium-Neorhizobium-Pararhizobium-Rhizobium* and *Massilia* (*P* < 0.05) (Supplementary Figure S4).

**FIGURE 2:**
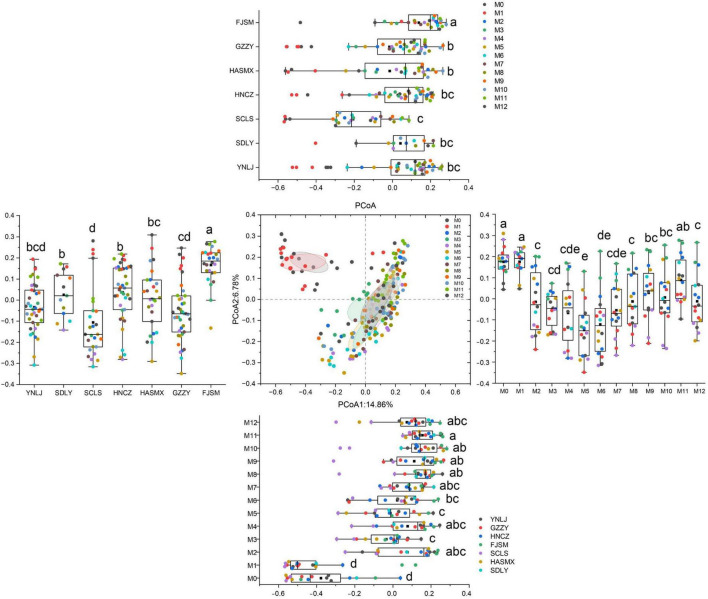
PCoA analysis of bacterial communities of different aged tobacco samples according to the classification of aging time, tobacco origin, tobacco grade and tobacco leaf collection site.

Principal Coordinate Analysis (PCoA) was performed to analyze the community clustering related to different factors (Figure 2). Regarding the source, the community of SCLS tobacco leaf could be differentiated from those of other sources along PCoA axis 1, and that of FJSM could be differentiated from those of other sources along PCoA axis 2. Concerning the aging time, the sample points shifted positively along PCoA axis 1 as the aging time increased. Among these, the communities at M0 and M1 exhibited significant differences compared to those at other time points.

### 3.2 Dynamics of volatile compounds in tobacco leaves during aging process

**FIGURE 3:**
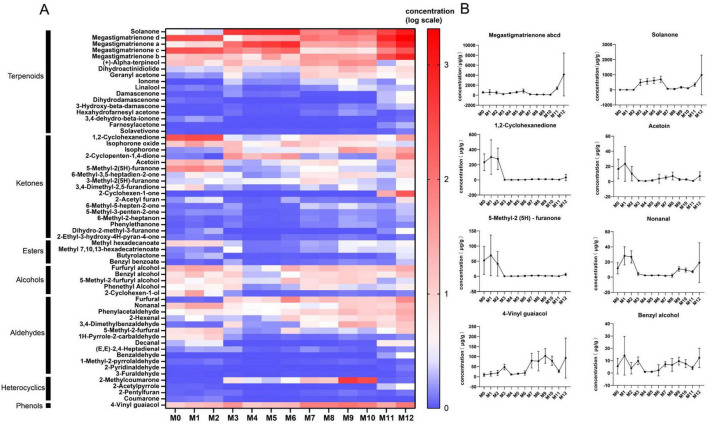
**(A)** Heatmap showing the content of neutral flavor components in tobacco leaves during 1 year of accelerated aging. Red color representing higher content and blue color representing lower content. **(B)** Line plot of the relative contents of the six volatile compounds that changed significantly by one-way ANOVA.

Figure 3 illustrates the composition of neutral flavor components in tobacco leaves across various aging stages. It reveals that, irrespective of regional variations, the changes in these components during aging exhibit remarkable consistency. For instance, megastigmatrienone (possessing a sweet tobacco aroma) and solanone (with a refreshing, carrot-like aroma) demonstrated a gradual rise as aging progresses. Additionally, 4-vinyl guaiacol (characterized by its distinctive fermentation aroma) experienced an upward trend throughout the aging procedure. Conversely, certain compounds including 1,2-cyclohexanedione and 5-methyl-2(5H)-furanone displayed a decline as aging time advanced. The concentration of nonanal initially decreased with time but subsequently rose after the M9 stage. These findings suggest that the observed alterations in aromatic components signify the positive impact of aging on tobacco leaf flavor, through enhancing the content of aroma-producing substances, ultimately leading to an improved overall quality of the tobacco.

### 3.3 Correlation between volatiles compound changes and bacterial community succession in tobacco leaves during aging process

**FIGURE 4:**
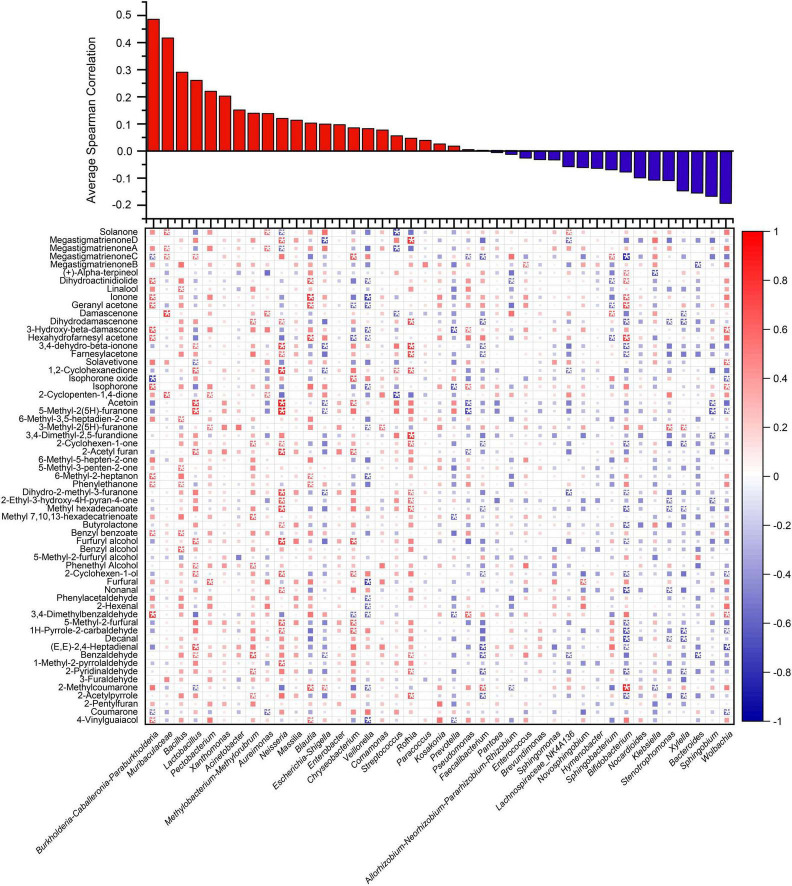
Spearman correlation heatmap illustrating associations between neutral flavor constituents of tobacco leaves undergoing accelerated aging and bacterial taxa (abundance > 0.1%). The heatmap is ordered by average correlation coefficient. Red indicates positive correlations, blue negative correlations, and * denotes *P* < 0.05.

Spearman relationship between neutral flavor components in tobacco leaves and bacterial species with an abundance exceeding 0.1% during the aging process is shown in Figure 4. The results showed that *Burkholderia-Caballeronia-Paraburkholderia*, *Muribaculaceae*, and *Bacillus* had a strong positive correlation with the flavor changes during the aging process of tobacco leaves, while *Bacteroides, Sphingobium*, and *Wolbachia* showed a strong negative correlation with the aging flavor of tobacco leaves. Consequently, *Burkholderia-Caballeronia-Paraburkholderia*, *Muribaculaceae*, and *Bacillus* are hypothesized to be key flavor-producing microorganisms during the aging process of tobacco leaves. Certain strains within the *Burkholderia-Caballeronia-Paraburkholderia* genus have been shown to benefit plants and function as plant growth-promoting bacteria (Luo et al., 2021). *Muribaculaceae* is predominantly found in the gastrointestinal tract of animals and constitutes an essential part of the intestinal microbial community (Zhu et al., 2024). *Bacillus* represents a group of microorganisms frequently utilized in fermentation processes, capable of producing enzymes and flavors. In this study, indigeneous *Bacillus* strains from tobacco leaves were isolated to investigate their potential impacts on tobacco leaf aging.

### 3.4 Isolation of *Bacillus* strains from tobacco leaves

**FIGURE 5:**
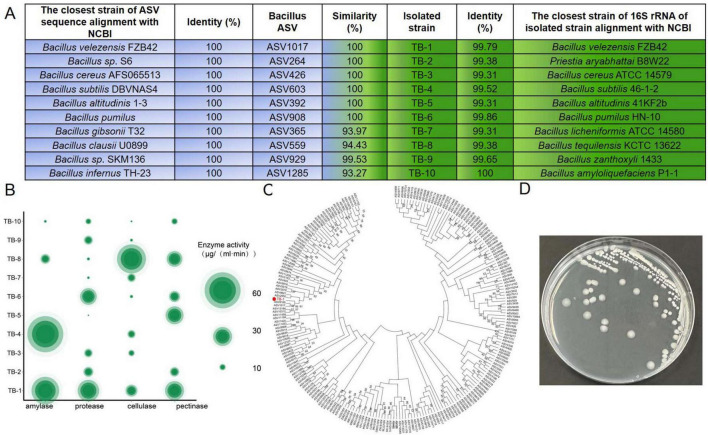
Isolation and identification of *Bacillus* species from aged tobacco leaves. **(A)** Matching results between the 16S rRNA sequences of isolated strains and the ASV sequences in 16S rRNA gene amplicon sequencing of tobacco leaves. **(B)** Amylase activity, protease activity, cellulase activity, and pectinase of isolated *Bacillus* species. **(C)** Evolutionary tree relationship between strain TB-1 and *Bacillus* ASV of aged tobacco leaves. **(D)** Plate colony morphology of *Bacillus velezensis* TB-1.

ASV sequences derived from amplicon sequencing were taxonomically annotated using the Genebank database. A total of 10 ASVs exhibited 100% nucleotide identity to *Bacillus* strains in the database (Figure 5A). Meanwhile, 26 strains were isolated from tobacco leaves using separation medium. Among them, the 16S rRNA gene sequences of 10 isolates could be matched with the 10 *Bacillus* ASVs, respectively, including 6 strains showing perfect homology. Taxonomic assignment via 16S rRNA profiling confirmed nine of 10 characterized isolates as *Bacillus* spp. (Figure 5A). These 10 strains were selected for further evaluation of enzyme production capacity.

The enzyme-producing properties of 10 *Bacillus* strains for amylase, protease, cellulase, and pectinase were compared. Among them, 4 strains had the ability to produce amylase, 9 strains could produce protease, 8 strains could produce cellulase, and 6 strains could produce pectinase (Figure 5B). Comparing the degradation ability of each strain, the results indicated that strain *Bacillus velezensis* TB-1 had the strongest comprehensive enzyme-producing ability, as it could produce all four enzymes. The enzyme activities of TB-1 were 45.8, 35.7, 19.6 and 31.5 μg/(mL ⋅ min), respectively (Figure 5B). A phylogenetic tree illustrates the relationship between *Bacillus velezensis* TB-1 and *Bacillus* ASV of aged tobacco leaves (Figure 5C). Colony morphology of *Bacillus velezensis* TB-1 on the plate is shown in Figure 5D.

### 3.5 Volatile compounds in tobacco leaves fermented by *Bacillus velezensis* TB-1

**FIGURE 6:**
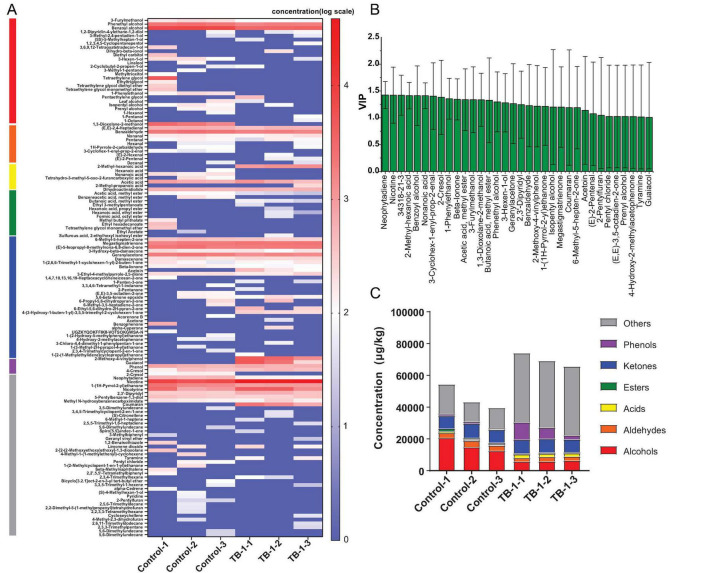
**(A)** Heatmap of volatile compound composition in *Bacillus velezensis* TB-1 fermented tobacco leaves. **(B)** Volatile compounds with VIP value of > 1 after PLS-DA analysis. **(C)** Relative content of various compounds (μg/kg).

The volatile compounds in tobacco leaves after *Bacillus velezensis* TB-1-augmented fermentation were detected by GC-MS. A total of 134 volatile compounds were detected in the samples after enhanced fermentation. Using the Student’s *t*-test, the content of eight volatile compounds in fermented tobacco leaves was found to have increased significantly compared to the control group, including 2-methyl-hexanoic acid, megastigmatrienone, 2-methoxy-4-vinylphenol, 4-(3-hydroxy-1-buten-1-yl)-3,5,5-trimethyl-2-cyclohexen-1-one, nicotine, 2,3’-dipyridyl, coumaran and butanoic acid, methyl ester. After PLS-DA analysis, 34 volatile compounds with VIP value of > 1 were obtained (Figure 6B). Combined with the results of VIP value screening and *t*-test, it is considered that these eight substances may have changed the flavor of tobacco leaves. As compared with the control group, the total relative content of volatile compounds in fermented tobacco leaves increased from 45,700.6 to 69,633.3 μg/kg (Figure 6). The content of other types of compounds, phenolic compounds, and acidic compounds has significantly increased. Among them, the nicotine content increased by 5 times from 6,762.3 to 35,669.5 μg/kg, making the flue gas more sufficient. However, volatile alcohols were significantly reduced, 1,3-Dioxolane-2-methanol decreased from 2,470.2 μg/kg to 0. At the same time, the content of Benzyl alcohol also decreased from 7,831.2 to 3,570.4 μg/kg. The fermented tobacco increased 1,193.3 μg/kg of 2-methyl-hexanoic acid in fruit cheese flavor, 249.8 μg/kg of buttery flavored acetoin, 462.4 μg/kg tobacco sweet flavor megastigmatrienone, 3,183.3 μg/kg of 2-methoxy-4-vinylphenol with intense spices, cloves and fermentation like aroma, which promoted the increase of aroma and comfort of flue-cured tobacco and the reduction of irritation. Changes in these compounds contribute to an increase in the aroma and comfort of flue-cured tobacco.

### 3.6 Sensory evaluation of tobacco leaves fermented by *Bacillus velezensis* TB-1

**FIGURE 7:**
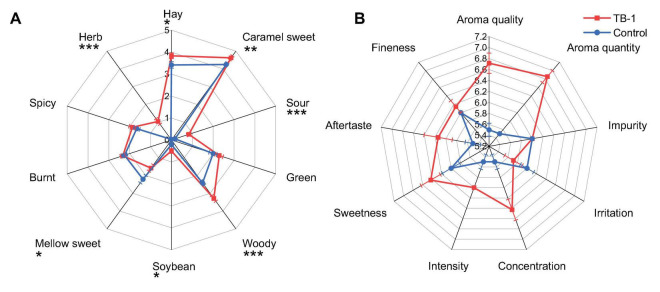
Sensory evaluation of *Bacillus velezensis* TB-1 fermented tobacco leaves. **(A)** Sensory odor radar chart of flavor characteristics in different treatment groups,“*,” significant (*P* ≤ 0.05); “**,” highly significant (*P* ≤ 0.01); “***,” very highly significant (*P* ≤ 0.001). **(B)** Radar chart of smoking evaluation scores of different treatment groups.

The difference in aroma style characteristics between TB-1 fermented tobacco leaves and the control group was determined through sensory evaluation, and the results are shown in Figure 7A. The results showed that there were significant differences in the aroma characteristics of tobacco leaves before and after fermentation. The control group of tobacco leaves contains a total of 7 aromas, including hay, caramel sweet, green, wood, burnt, mellow sweet, and spicy. Compared with the control group, the fermented tobacco group increased three aromas, including sour, soybean, and herb. The total aroma score of fermented tobacco is higher than that of the control group, indicating that the aroma of fermented tobacco leaves is richer. Fermented tobacco significantly increases the hay, caramel sweet, wood, and sour, while reducing the mellow sweet aroma.

Through a triangle test involving the addition of compounds at corresponding levels, it was observed that nicotine and 2-methoxy-4-vinylphenol exert a highly significant influence on the flavor of tobacco leaves (Table 1). The increase in the concentrations of 2-methyl-hexanoic acid, megastigmatrienone, and coumaran led to notable variations in the tobacco leaf flavor. However, the inclusion of butanoic acid, methyl ester, 4-(3-hydroxy-1-buten-1-yl)-3,5,5-trimethyl-2-cyclohexen-1-one, and 2,3’-dipyridyl did not exhibit significant differences in the flavor of tobacco leaves compared to the control group.

**TABLE 1:** Triangle test for the addition of 8 significantly increased volatile compounds.

No.	Added volatile compounds	N^a^	significant^b^
1	2-Methyl-hexanoic acid	8	*
2	Butanoic acid, methyl ester	4	
3	Megastigmatrienone	8	*
4	4-(3-Hydroxy-1-buten-1-yl)-3,5,5-trimethyl-2-cyclohexen-1-one	3	
5	2-Methoxy-4-vinylphenol	11	***
6	Nicotine	12	***
7	2,3′’-Dipyridyl	4	
8	Coumaran	9	**
			

^a^Number of correct judgments from 12 assessors evaluating the odor difference by the triangle test. ^b^“*,” significant (*p* ≤ 0.05); “**,” highly significant (*p* ≤ 0.01); “***,” very highly significant (*p* ≤ 0.001).

The tobacco leaves fermented by *Bacillus velezensis* TB-1 and the uninoculated control tobacco leaves were rolled into tobacco sticks. According to the GB 5,606.4–2005 sensory evaluation protocol, seven trained evaluators assessed evaluate the treated tobacco leaves across three dimensions: aroma characteristics (including aroma quality, aroma quantity, impurity), taste characteristics (sweetness, aftertaste, irritation), and smoke characteristics (concentration, intensity, and fineness). The control sample was on a 9-point scale, and the fermented sample was treated with sensory deviation reference method. The results showed that TB-1 fermentation treatment improved the aroma characteristics of tobacco leaves, and the quality and quantity of aroma were improved. In terms of taste characteristics, both sweetness and aftertaste increase, resulting in a sweeter taste, less pungency and a better aftertaste (Figure 7B). The smoke characteristics have also been improved, with increased concentration and moderate intensity and finesse. Overall, the TB-1 group samples were characterized by aroma penetration, increased quantity and concentration, and had a roasted sweetness. In summary, inoculation with TB-1 can effectively improve the characteristics of flue-cured tobacco, with better aroma, smoke and sweet taste.

## 4 Discussion

Just like other fermented foods, tobacco leaf aging is an effective process to improve the quality of tobacco leaves. The study on microbial improvement of tobacco leaf quality was first reported in Zhang et al. (2022). The study showed that microorganisms can increase the aroma components of tobacco leaves, especially *Bacillus* and micrococcus, among the tested microbial strains. In recent years, researches on the improvement of tobacco leaf flavor by tobacco aging mostly focused on the microorganisms in the natural aging process of tobacco leaves (Chen S. et al., 2024). Zhou et al characterized the changes of microbial community and diversity in tobacco leaves aged for 24 months and believed that the microbial community in tobacco leaves aged had significant spatiotemporal heterogeneity (Zhou et al., 2020). Wu et al. analyzed aged and unaged tobacco leaves by microbiome and concluded that *Acinetobacter*, *Sphingosphinomonas* and *Asperasperus* are important contributors to the volatile and non-volatile diferential compounds (DCs) of tobacco leaves (Wu et al., 2022). Mao et al. screened *Bacillus subtilis* and fermented low-grade tobacco leaves with improved sensory aroma and increased aroma (Mao et al., 2022). Li et al. isolated *Bacillus mojavensis* from the surface of tobacco leaf. After fermentation, it showed good effect in harmonizing chemical components of tobacco leaf and increasing the content of flavoring substances (Li et al., 2023). The bacterial succession pattern of tobacco leaf aging in this study was similar to that of Zhou and Wu, and the abundance of *Pseudomonas* and *Sphingosphinomonas* was stable, and *Acinetobacter* and *Methylbacterium* increased slightly with aging time. But the difference is that there are a large number of unidentified bacteria in the bacterial community of tobacco leaves in this study. In this study, the abundance of *Enterobacteriaceae* was low and did not change significantly during the aging cycle of tobacco leaves. In this study, 16S rRNA sequencing and GC-MS technology were used to study the bacterial communities and neutral flavor components on the surface of 16 different tobacco leaves from 7 production areas in China. In particular, fermentation microorganisms were selected through the correlation between neutral flavor components and bacterial communities for tobacco leaf aging Through correlation analysis, it was found that *Bacillus* had strong positive correlation with neutral flavor components of aged tobacco leaves. Therefore, it was speculated that *Bacillus* is the key flavor-producing microorganism in accelerating the aging of tobacco leaves, which is similar to the previous results.

In order to further verify the effect of *Bacillus* on fermented tobacco leaves, a strain of *Bacillus* was isolated and cultured to be used in the fermentation of low-grade tobacco leaves. The results showed that compared with the control group, the volatile compounds of flue-cured tobacco treated with *Bacillus* had obvious changes. The content of neophytadiene decreases and the nicotine content increases, making the smoke more abundant. The neutral flavor components such as megastigmatrienone, acetoin, 2-methoxy-4-vinylphenol, nonanal and 4-(3-hydroxy-1-buten-1-yl)-3,5,5-trimethyl-2-cyclohexen-1-one increased, which could increase the hay, sweet and woody flavor of tobacco leaves. The changes in flavor of tobacco leaves after TB-1 fermentation are similar to those of aging tobacco leaves, such as a significant increase in the content of megastigmatrienone and 2-methoxy-4-vinylphenol. According to research, megastigmatrienone is a compound positively correlated with smoking evaluation, and the increase of megastigmatrienone in fermented tobacco leaves may improve the quality of smoking evaluation (Cao et al., 2012; Yao et al., 2022). Multiple studies have shown that *Bacillus* can promote the production of 2-methoxy-4-vinylphenol, which is consistent with the results of this study (Mishra et al., 2014; Wang et al., 2025). Based on this, it is speculated that 4-vinylguaiacol in this study is likely produced through TB-1 fermentation, which enhances the woody aroma of tobacco leaves. In addition, more 2-methyl-hexanoic acid is produced, which gives the tobacco a cheesy flavor and ultimately improves the flavor of the tobacco. According to research (Gong et al., 2023), the action of starch degrading bacteria on tobacco leaves can increase the Maillard reaction products, and it is speculated that the increase of these compounds may be attributed to the fact that *Bacillus* TB-1 can degrade starch and produce Maillard reaction products, which can enhance the flavor of tobacco leaves. In addition to these similarities, the special feature of this study is that the *Bacillus* TB-1 obtained *in situ* from tobacco leaves can produce a large amount of phenolic compounds, which improves the flavor of tobacco leaves (Chen X. F. et al., 2024). In this study, only single bacteria were used for pure culture fermentation, and the fermentation conditions were not optimized. In the future, it is necessary to further study the fermentation conditions of tobacco leaves and try to construct multi-strain co-culture.

## 5 Conclusion

A correlation analysis of the dynamic changes in bacterial communities and neutral flavor compounds during accelerated aging of tobacco from seven Chinese regions revealed that *Bacillus* was a key genus driving aroma production. This genus exhibited positive correlations with various volatile compound alterations in tobacco leaves. Using the pure culture, *Bacillus velezensis* TB-1, a strain with superior enzyme-producing capacity, was isolated from tobacco leaves. The fermentation of tobacco leaves by *Bacillus velezensis* TB-1 increased the levels of sweet and woody flavor compounds, including 2-methyl-hexanoic acid, 2-methoxy-4-vinylphenol, acetoin, and megastigmatrienone, thereby significantly enhancing the flavor. In terms of sensory evaluation, the fermented tobacco leaves enhanced sensory indices, including aroma intensity, sweetness, aroma quality, intensity, concentration, and aftertaste. These findings offer significant theoretical insights into the changes in bacterial communities and microbial-enhanced fermentation of flue-cured tobacco during the aging process.

## Data Availability

The datasets presented in this study can be found in online repositories. The names of the repository/repositories and accession number(s) can be found in the article/Supplementary material.

## Footnotes

1
http://www.ncbi.nlm.nih.gov/BLAST/
