# Supplementary material for: Isolation of indigenous Bacillus velezensis from aging tobacco leaves for improving the flavor of flue-cured tobacco
Source: Front Microbiol. 2025 Jul 16;16:1623279. doi: 10.3389/fmicb.2025.1623279 (PMC12307346; doi:10.3389/fmicb.2025.1623279)
Supplement: Supplementary file 1 [file Supplementary_file_1.docx]

**Supplementary files:**

**Isolation of indigenous *Bacillus*** ***velezensis* from aging tobacco leaves for improving the flavor of flue-cured tobacco**

Xiao-Jie Shan^a,#^, Yue Yang^b,#^, Yi-Fan Zhang^b,#^, Qiang Xu^b^, Chen-Lin Miao ^b^, Lifeng Jin^c^, Feng Li^c^, Sheng-Bing Yang^a^, Xiao-Juan Zhang^a,e^, Li-Juan Chai^a,e^, Jin-Song Gong^f^, Jin-Song Shi^f^, Zhen-Ming Lu^a,e,*^, Zong-Yu Hu^b,*^, Zheng-Hong Xu^a,d,*^

^a^ *Key Laboratory of Industrial Biotechnology of Ministry of Education, School of Biotechnology, Jiangnan University, Wuxi 214122, China*

^b^ *China Tobacco Jiangsu Industrial Co., Ltd, Nanjing 210019, China*

^c^ *Zhengzhou Tobacco Research Institute of CNTC, Zhengzhou 450001, China*

^d^ *Innovation Center for Advanced Brewing Science and Technology, College of Biomass Science and Engineering, Sichuan University, Chengdu 610065, PR China*

^e^ *National Engineering Laboratory of Cereal Fermentation and Food Biomanufacturing, Jiangnan University, Wuxi 214122, China*

^f^ *School of Life Science and Health Engineering, Jiangnan University, Wuxi 214122, China*

#These authors contributed equally to this work.

* Corresponding author. *E-mail address*: zmlu@jiangnan.edu.cn (Z.M. Lu); huzy707@sina.com (Z. Hu); zhenghxu@scu.edu.cn (Z.H. Xu);

**Table S1.** Tobacco sample information

| No | province | | region | varieties |
| --- | --- | --- | --- | --- |
| 1 | | Yunnan (YN) | Lijiang (LJ) | Yunyan87 |
| 2 | | Guizhou (GZ) | Zunyi (ZY) | Yunyan87 |
| 3 | | Hunan (HN) | Chenzhou (CZ) | Yunyan87 |
| 4 | | Sichuan (SC) | Liangshan (LS) | Yunyan87 |
| 5 | | Fujian (FJ) | Sanming (SM) | CB-1 |
| 6 | | Henan (HA) | Sanmenxia (SMX) | Qinyan96 |
| 7 | | Shandong (SD) | Linyi (LY) | Zhongyan Texiang301 |


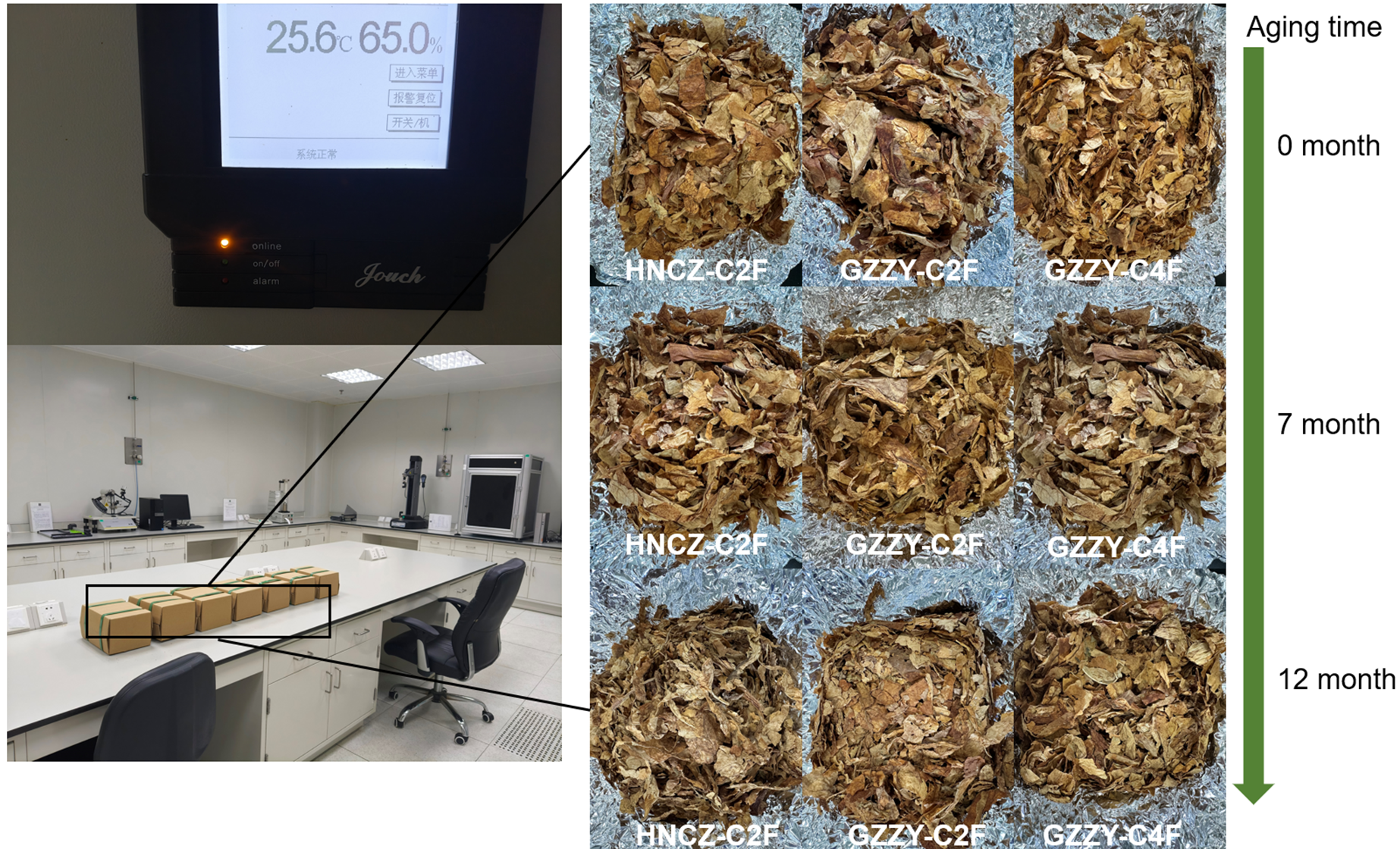


**Figure S1** Accelerated aging of tobacco leaves was performed in a room with controlled environmental condition (temperature, 25℃-30℃; humidity, 60%-65%).


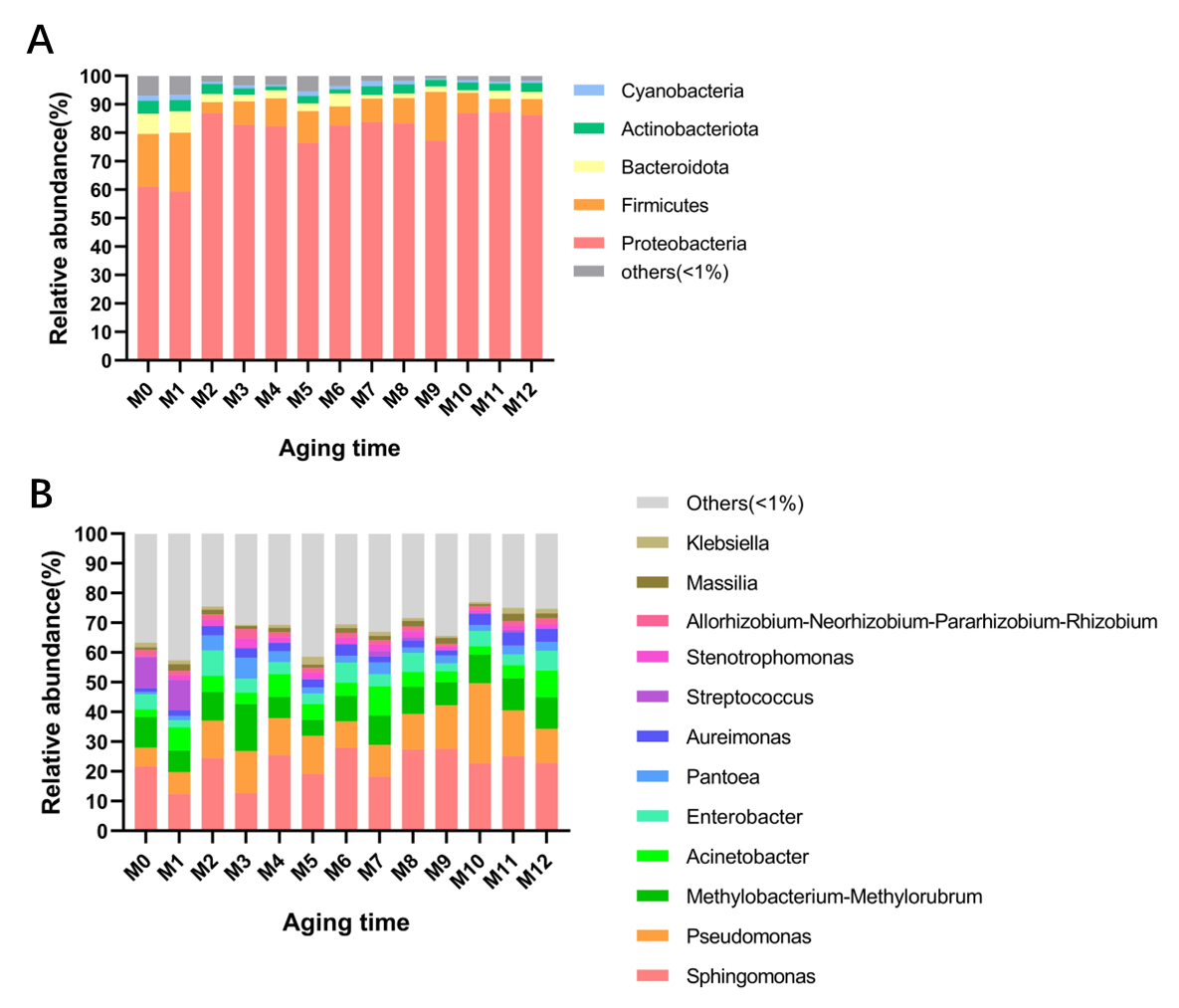


**Figure S2** Bacterial community succession of tobacco leaf during the accelerated aging process at the phylum level (A) and the genus level (B). Species with an unannotated classification and a relative abundance below 0.5% in the sample were grouped into the ‘others’ category.


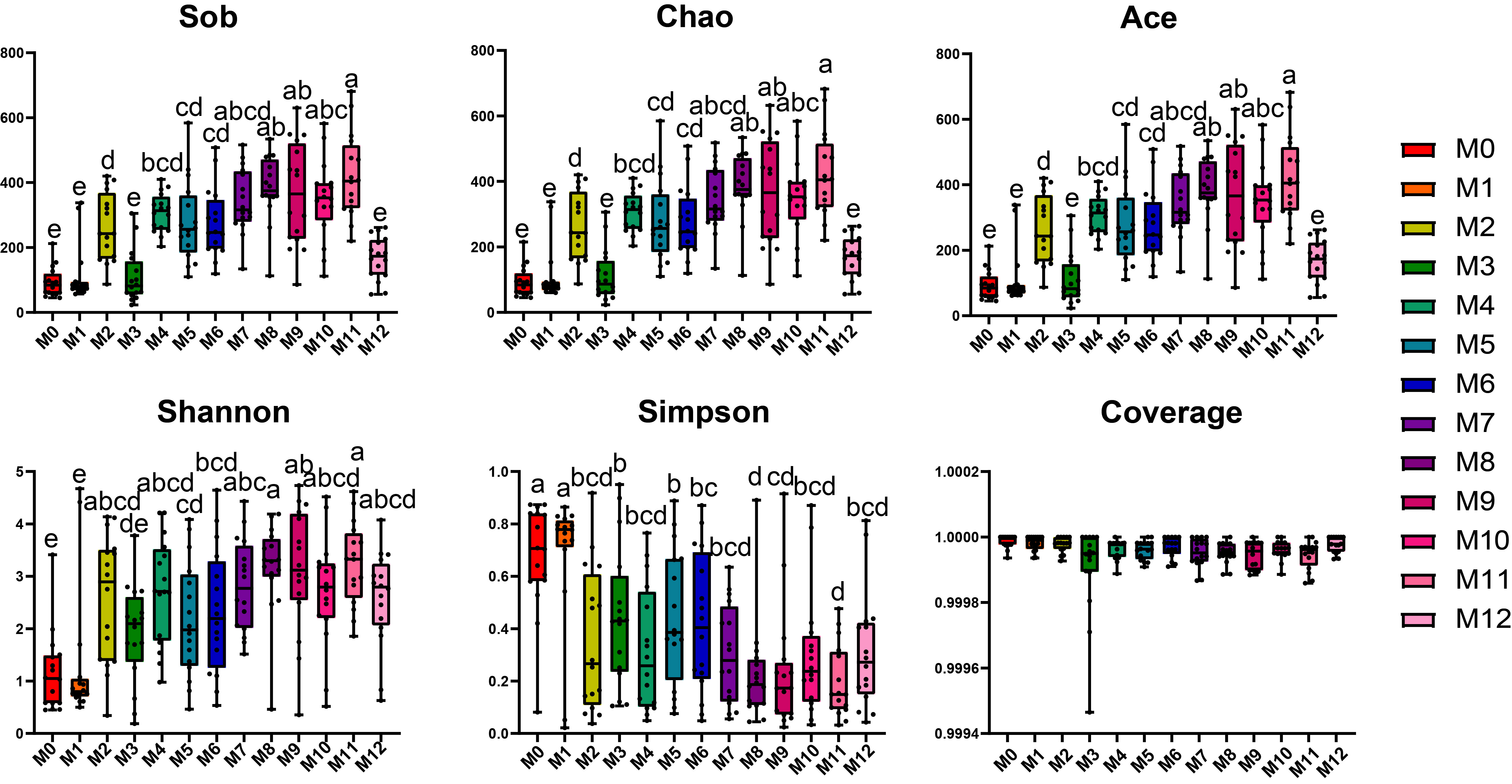


**Figure S3** α-Diversity index of bacterial community in tobacco leaf from the 1st to 13th stages of accelerated aging. The data are presented as the mean±standard deviation (SD) and the groups were accompanied by one-way analysis of variance (ANOVA). Duncans’ test was performed for determining the significance. Columns marked with different letters possess values of significantly difference (*P* < 0.05).


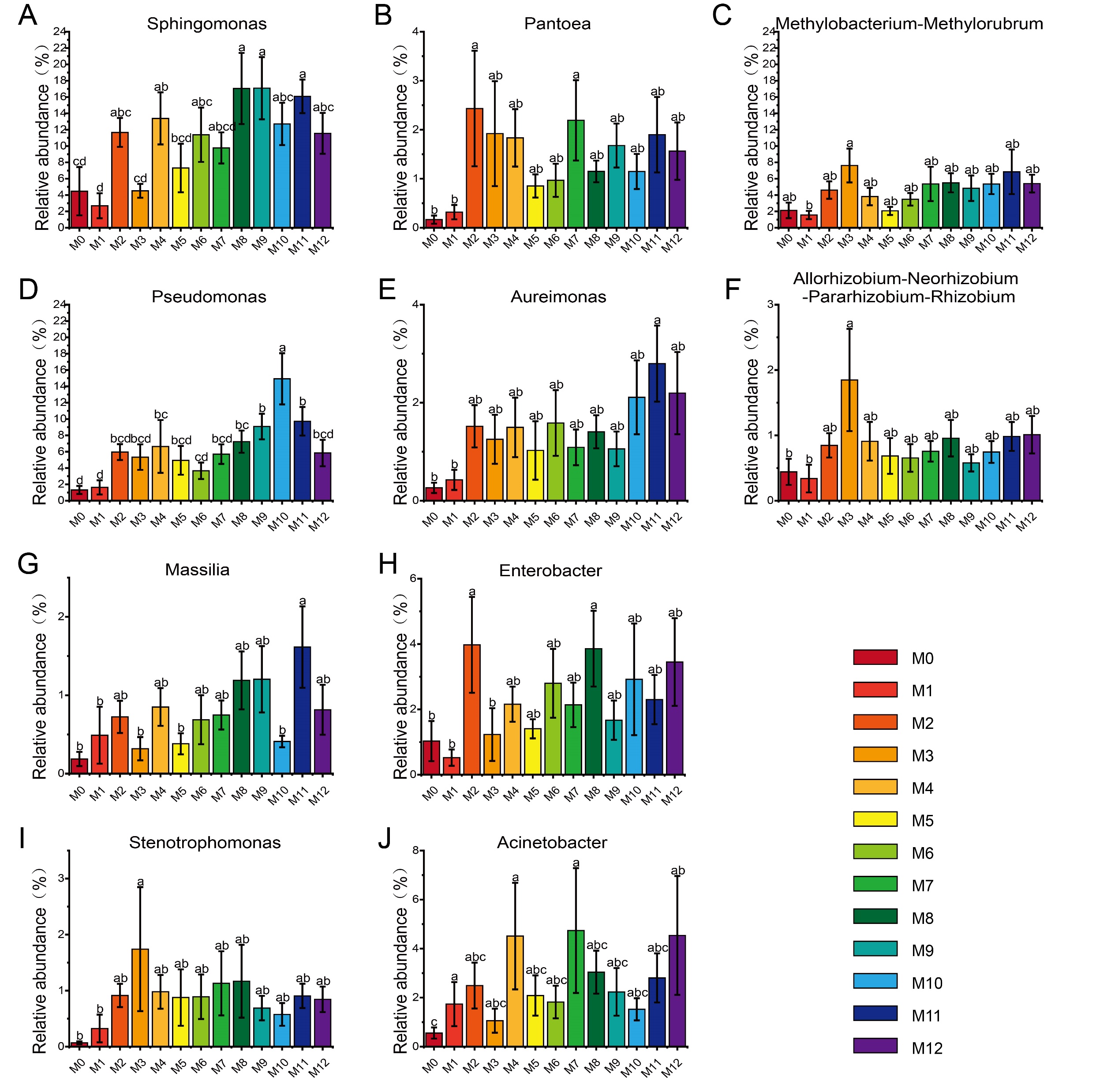


**Figure S4** Dynamics of average relative abundance of the top 10 genera in the bacterial community of tobacco leaves. The data are presented as the mean±standard deviation (SD) and the groups were accompanied by one-way analysis of variance (ANOVA). Duncans’ test was performed for determining the significance. Columns marked with different letters possess values of significantly difference (*P* < 0.05).
